# MODUL cohort 2: an adaptable, randomized, signal-seeking trial of fluoropyrimidine plus bevacizumab with or without atezolizumab maintenance therapy for *BRAF*^*wt*^ metastatic colorectal cancer☆

**DOI:** 10.1016/j.esmoop.2022.100559

**Published:** 2022-08-24

**Authors:** J. Tabernero, A. Grothey, D. Arnold, A. de Gramont, M. Ducreux, P. O’Dwyer, A. Tahiri, F. Gilberg, N. Irahara, H.-J. Schmoll, E. Van Cutsem

**Affiliations:** 1 Vall d’Hebron Hospital Campus and Institute of Oncology (VHIO), IOB-Quiron, UVic-UCC, Barcelona, Spain; 2 West Cancer Center, Germantown, USA; 3 Asklepios Tumorzentrum Hamburg, AK Altona, Hamburg, Germany; 4 Franco-British Hospital, Levallois-Perret, France; 5 Gustave Roussy, Université Paris Saclay, Villejuif, France; 6 Abramson Cancer Center, University of Pennsylvania, Philadelphia, USA; 7 F. Hoffmann-La Roche Ltd, Basel, Switzerland; 8 Martin-Luther-University, Halle, Germany; 9 University Hospitals Gasthuisberg, Leuven and KU Leuven, Leuven, Belgium

**Keywords:** atezolizumab, bevacizumab, metastatic colorectal cancer, PD-L1

## Abstract

**Background:**

MODUL is an adaptable, signal-seeking trial designed to test novel agents in predefined patient subgroups in first-line metastatic colorectal cancer (mCRC).

**Patients and methods:**

Patients with measurable, unresectable, previously untreated mCRC received induction with ≤8 cycles of FOLFOX + bevacizumab followed by randomization to maintenance treatment comprising control [fluoropyrimidine (FP)/bevacizumab: 5-fluorouracil 1600-2400 mg/m^2^ 46-h intravenous (i.v.) infusion day 1 q2 weeks plus leucovorin 400 mg/m^2^ 2-h infusion i.v. day 1 q2 weeks or capecitabine 1000 mg/m^2^ b.i.d. orally days 1-14 every 21 days; bevacizumab 5 mg/kg 15-30-min i.v. infusion q2 weeks] or experimental treatment in one of four biomarker-driven cohorts. In patients with *BRAF* wild-type (*BRAF*^*wt*^) tumors (cohort 2), experimental treatment was FP/bevacizumab + atezolizumab (800 mg 60-min i.v. infusion q2 weeks). Primary efficacy endpoint was progression-free survival (PFS; intent-to-treat population). Enrollment is complete; efficacy and safety findings from cohort 2 are presented.

**Results:**

Four hundred and forty-five patients with *BRAF*^*wt*^ mCRC were randomized (2 : 1) to maintenance in cohort 2. At a median follow-up of 10.5 months, PFS outcome hypothesis was not met [hazard ratio (HR) 0.92; 95% confidence interval (CI) 0.72-1.17; *P* = 0.48]; overall survival (OS) was immature. At a median follow-up of 20.3 months (2-year survival follow-up), PFS benefit was also not met (HR 0.95; 95% CI 0.77-1.18; *P* = 0.666); OS HR with nearly two-thirds of patients with events was 0.83 (95% CI 0.65-1.05; *P* = 0.117). No new safety signals were identified. The most common grade ≥3 treatment-emergent adverse events (TEAEs) for experimental versus control arms were hypertension (6.1% versus 4.2%), diarrhea (3.1% versus 2.1%), and palmar-plantar erythrodysesthesia syndrome (1.0% versus 2.5%). Four patients experienced TEAEs with fatal outcome, two were study treatment-related: hepatic failure (experimental arm) and large intestine perforation (control arm; bevacizumab-related).

**Conclusions:**

Adding atezolizumab to FP/bevacizumab as first-line maintenance treatment after FOLFOX + bevacizumab induction for *BRAF*^*wt*^ mCRC did not improve efficacy outcomes.

## Highlights

• MODUL is an adaptable, signal-seeking trial of novel combinations in predefined subgroups of patients with mCRC.
• In cohort 2 (*BRAF*^*wt*^ mCRC), adding atezolizumab to FP/bevacizumab (bev) first-line maintenance therapy did not improve PFS or OS versus FP/bev.
• ORR and DCR were numerically but not statistically significantly higher with atezolizumab + FP/bev versus FP/bev.
• The safety profile of atezolizumab + FP/bev was consistent with previous findings with no new safety signals identified.
• Findings from MODUL cohort 2 indicate that immunotherapy has limited efficacy in patients with microsatellite stable mCRC.

## Introduction

In patients with metastatic colorectal cancer (mCRC), molecular screening approaches and new biomarkers are required to fully characterize tumors and identify those most likely to benefit from specific therapies.^1^^,^^2^ Tumor cell pathways and microenvironments that play significant roles in disease prognosis and response to therapeutic agents vary widely between patients, and represent potential opportunities to tailor treatment selection to the individual and optimize therapeutic outcomes. Patients typically receive first-line induction therapy to achieve disease control followed by de-escalated maintenance therapy to delay disease progression and limit cumulative toxicities.^3^, ^4^, ^5^, ^6^, ^7^, ^8^ Of relevance for clinical research, the first-line maintenance setting can be used to identify a signal of activity with conceivably less tumor heterogeneity than in later lines.

**Figure 1:**
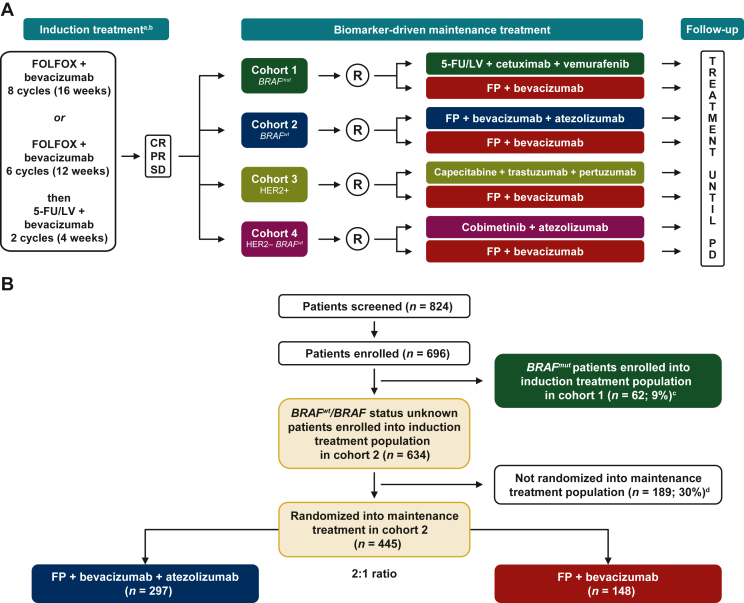
MODUL study design (A), and patient disposition (cohorts 1 and 2) (B). 5-FU/LV, 5-fluorouracil/leucovorin; CR, complete response; FOLFOX, 5-fluorouracil/leucovorin + oxaliplatin; FP, fluoropyrimidine; HER2, human epidermal growth factor receptor 2; i.v., intravenous; mCRC, metastatic colorectal cancer; PD, progressive disease; PR, partial response; R, randomization; SD, stable disease. ^a^Key eligibility criteria: histologically confirmed mCRC; measurable, unresectable disease (RECIST version 1.1); no prior chemotherapy for mCRC; age ≥18 years; Eastern Cooperative Oncology Group performance status ≤2. ^b^Patients with disease progression following induction treatment received further treatment at the discretion of their physician. ^c^*BRAF* mutations were V600. ^d^Main reasons for not being randomized into maintenance treatment population: disease progression, surgery, violation of criteria. **Cohort2: Design details** First randomization: August 2015; last patient randomization: 9 November 2016. Clinical cut-off date: 31 May 2017 (primary analysis); 31 May 2019 (2-year analysis). *Experimental arm* FP (5-FU/LV or capecitabine): 5-FU 1600-2400 mg/m^2^ administered via 46-h i.v. infusion on day 1 of every 2-week cycle and LV 400 mg/m^2^ administered via a 2-h infusion i.v. on day 1 of every 2-week cycle; or capecitabine 1000 mg/m^2^ twice-daily by mouth given on days 1-14 followed by a 1-week treatment break. Patients receiving capecitabine were directed to take each dose within 30 min after meals. Bevacizumab: 5 mg/kg via 15-30-min i.v. infusion every 2-week cycle. Bevacizumab was prepared and administered in accordance with local prescribing information. Atezolizumab: atezolizumab was administered at a fixed dose of 800 mg via 60-min i.v. infusion every 2-week cycle. Premedication was not indicated for the first dose of atezolizumab. Premedication was allowed for cycles ≥2 at the discretion of the treating physician. *Control arm* FP (5-FU/LV or capecitabine): dose and schedule were according to local labeling, where applicable, or otherwise were determined as per the investigator’s discretion. Administration was according to local prescribing information. Patients receiving capecitabine were directed to take each dose within 30 min after meals. Bevacizumab: 5 mg/kg via 15-30-min i.v. infusion every 2-week cycle. Bevacizumab was prepared and administered in accordance with local prescribing information. Premedication was not considered warranted.

The MODUL study (ClinicalTrials.gov: NCT02291289) is a highly adaptable, signal-seeking platform that allows testing of novel combinations in predefined molecular subgroups of patients with a common control arm of standard maintenance in first-line mCRC. MODUL follows an umbrella design,^9^ wherein patients with mCRC receive first-line induction treatment with 5-fluorouracil/leucovorin (5-FU/LV) and oxaliplatin (FOLFOX) plus bevacizumab, the standard of care in many countries for the treatment of mCRC when combined with fluoropyrimidine (FP)-based chemotherapy. Following induction treatment, patients are assigned to one of multiple maintenance treatment cohorts based on their cancer’s biomarker profile (Figure 1A). The rationale for the maintenance treatment regimens evaluated in the MODUL study has been discussed in detail elsewhere.^10^

Cohort 2 of MODUL, which compares the efficacy and safety of maintenance treatment with an FP (either capecitabine or 5-FU/LV) and bevacizumab with or without atezolizumab in patients with mCRC without *BRAF* mutation in their primary tumor sample, represents a group of patients with mCRC for whom a predictive biomarker has not been identified. Approximately 95% of patients have mCRC that is mismatch repair proficient and microsatellite stable (MSS).^11^ Single-agent programmed cell death-ligand 1 (PD-L1) inhibitors have demonstrated no meaningful activity in MSS mCRC.^11^ However, combining vascular endothelial growth factor (VEGF) inhibitors (e.g. bevacizumab) with PD-L1 inhibitors like atezolizumab may reverse VEGF-mediated immunosuppression, support dendritic cell maturation, and promote T-cell infiltration into the tumor, as supported by published preclinical evidence.^12^^,^^13^ Consequently, atezolizumab was selected for combination with standard of care FP + bevacizumab based on preclinical and clinical evidence supporting its antitumor activity in mCRC and in patients not selected for *BRAF*-mutated mCRC.

The MODUL study was initiated in April 2015, starting with two maintenance treatment cohorts, with additional cohorts being added based on the availability of research data and new drugs as part of the planned evolution of the trial. The trial had four cohorts—three of which will be reported at a later date (cohorts 1, 3, and 4). The focus of this paper is cohort 2, which had the highest recruitment rate, in which patients with *BRAF* wild-type (*BRAF*^*wt*^) mCRC received maintenance treatment with either an FP + bevacizumab + atezolizumab (experimental arm) or FP + bevacizumab (control arm).

## Patients and methods

### Study design and participants

Patients were recruited in Europe, Asia, Africa, and South America (Supplementary Table S1, available at https://doi.org/10.1016/j.esmoop.2022.100559). Eligible patients were adults (aged ≥18 years) with histologically confirmed, measurable, unresectable mCRC (RECIST version 1.1), no prior chemotherapy for metastatic disease, an Eastern Cooperative Oncology Group performance status (ECOG PS) of ≤2, and ≥16 weeks of life expectancy at the time of study entry. For cohort 2, patients were required to have primary tumors with no mutation at the V600 codon (central assessment using cobas® 4800 *BRAF* V600 Mutation Test; Roche Diagnostics International AG, Rotkreuz, Switzerland) of the *BRAF* gene (*BRAF*^*wt*^ disease) or to have had no successful *BRAF* mutational testing (for technical reasons).

Safety and efficacy data were monitored by an independent data monitoring committee (iDMC). The iDMC was responsible for overseeing interim evaluations of safety and, as necessary, response in each of the study cohorts to ensure that accrual to any cohort not demonstrating risk−benefit balance was terminated early.

All procedures carried out in MODUL were in accordance with the International Conference on Harmonisation E6 Guideline for Good Clinical Practice and the principles of the Declaration of Helsinki, or the laws and regulations of the country in which the research is conducted. All patients provided written informed consent to participate in the study. The study protocol, informed consent forms, any information to be given to the patient, and relevant supporting information were all reviewed and approved by the Institutional Review Board/Ethics Committee before the study was initiated. The study protocol is available at https://clinicaltrials.gov/ProvidedDocs/89/NCT02291289/Prot_000.pdf.

## Randomization and masking

Following a 28-day screening period that included submission of a primary tumor sample for biomarker analysis, patients eligible for the study were enrolled, and received eight cycles of induction treatment over ∼4 months (the induction treatment phase). Within 3 weeks of completing induction treatment, patients who had not progressed and whose disease was not assessed as resectable were assigned to a maintenance treatment cohort based on the biomarker analysis results from their primary tumor sample. Patients whose primary tumor was *BRAF*^*wt*^ and patients whose primary tumor biomarker status was unknown were assigned to cohort 2 for maintenance treatment.

Following assessment of cohort-specific eligibility, patients were randomized to either experimental or control treatment by an independent interactive voice or web-based response system. Randomization occurred in a 2 : 1 ratio (experimental : control) and was stratified by geographical region (Europe, Americas, Africa, or Asia) and by patient response after induction treatment [complete response (CR)/partial response (PR) versus stable disease (SD)]. The randomization was carried out using a dynamic randomization algorithm.

## Procedures

First-line induction treatment was specified to be either eight 2-week cycles of FOLFOX with bevacizumab or six 2-week cycles of FOLFOX/bevacizumab, followed by two 2-week cycles of 5-FU/LV/bevacizumab. For maintenance, patients randomized to the control arm of cohort 2 received FP and bevacizumab in 2- or 3-week treatment cycles, depending on the FP used [5-FU 1600-2400 mg/m^2^ 46-h intravenous (i.v.) infusion and LV 400 mg/m^2^ 2-h i.v. infusion plus bevacizumab 5 mg/kg 15-30-min i.v. infusion on day 1 every 2 weeks or capecitabine 1000 mg/m^2^ twice-daily orally on days 1-14 every 21 days plus bevacizumab 5 mg/kg 15-30-min i.v. infusion every 2 weeks]. Patients randomized to the experimental arm received this regimen combined with atezolizumab in 2-week treatment cycles (800 mg 60-min i.v. infusion every 2 weeks; Figure 1A).

## Outcomes

The primary efficacy endpoint was progression-free survival (PFS), defined as time from randomization to maintenance treatment until disease progression according to RECIST (version 1.1; as per investigator assessment) or death from any cause, whichever occurred first.^14^ Secondary efficacy endpoints were overall survival (OS), overall response rate (ORR), disease control rate (DCR), time to treatment response (TTR), duration of response (DoR), and change in ECOG PS. PFS and OS analyses were repeated for the following predefined subgroups: age (<65 versus ≥65 years); sex (male versus female); region (Europe versus rest of the world); tumor response at the end of the induction treatment phase (SD versus CR/PR); baseline ECOG PS (0 versus 1/2), American Joint Committee on Cancer/Union for International Cancer Control stage at diagnosis (I/II/III versus IV); prior systemic adjuvant therapy (yes versus no); number of metastatic sites at baseline (<2 versus ≥2); liver metastatic sites at baseline (yes versus no); cancer type (colon versus rectal); tumor colon location (right versus left); and initial diagnosis (synchronous versus metachronous). Although microsatellite instable (MSI) and *BRAF*^*mut*^ patients were required to enter in other cohorts, predefined tumor biomarker subgroups included: *RAS* gene status [wild-type (*RAS*^*wt*^) versus mutant (*RAS*^*mut*^)]; microsatellite stability status (MSS versus MSI); *RAS* status (*RAS*^*wt*^ versus *RAS*^*mut*^) for MSS patients; tumor colon location (right versus left) for *RAS*^*wt*^ patients; tumor colon location (right versus left) for *RAS*^*mut*^ patients; tumor colon location (right versus left) for MSS patients.

Treatment-emergent adverse events (TEAEs) and serious adverse events (SAEs) were summarized by Medical Dictionary for Regulatory Activities (MedDRA) primary System Organ Classes and MedDRA Preferred Terms, grade, relationship to study treatment, and, for TEAEs during the maintenance treatment phase only, by events leading to dose modifications or death. TEAEs of special interest reported in electronic case report forms were summarized by system organ class, preferred terms, severity, relatedness, and seriousness. Laboratory data were classified according to National Cancer Institute Common Terminology Criteria for Adverse Events (version 4.0) where possible. All deaths were summarized with reason for death and by study period.

## Statistical analysis

For each maintenance cohort in MODUL (cohorts, 1, 2, 3, and 4), sample size was calculated based on assumptions of the primary study endpoint (PFS) within the cohort population and a primary analysis was conducted once the target number of PFS events had been reached. In cohort 2, to demonstrate an increase in median PFS from 7.5 months (control arm) to 11.5 months (experimental arm) corresponding to a hazard ratio (HR) of 0.65, a total of 405 randomized patients were required to observe 259 PFS events with 90% power and a two-sided significance level of 5%. The primary analysis was scheduled to be carried out when the target number of PFS events (*n* = 259) had been reached, which was estimated to occur ∼22 months after the first patient was randomized (clinical cut-off date: 31 May 2017). However, at the time of the primary clinical cut-off date (31 May 2017), a total of 292 PFS events had occurred. A further 2-year follow-up analysis was scheduled to take place once the 2-year survival follow-up from the primary cut-off date was complete (clinical cut-off date: 31 May 2019).

PFS was compared between experimental and control arms using an unstratified log-rank test and was estimated for each arm using Kaplan–Meier product-limit method estimates. The Brookmeyer–Crowley method was used to compute 95% confidence intervals (CIs).^15^ The estimated HR (FP + bevacizumab + atezolizumab versus FP + bevacizumab) and its corresponding 95% CI were obtained from an unadjusted Cox model with treatment as the single covariate. In the OS analysis, patients who were still alive at the time of analysis (clinical cut-off) and patients who were lost to follow-up were censored at their last clinical assessment date. ORR and DCR were summarized and presented along with 95% Clopper–Pearson CIs. The secondary time-to-event endpoints were analyzed by the same methods and at the same time as the primary endpoint. The above efficacy outcomes were evaluated using RECIST (version 1.1).

## Results

In total, 824 patients were screened, 696 of whom were enrolled in the study, which ran from 17 April 2015 to 24 March 2021. Of the 634 patients with *BRAF*^*wt*^*/BRAF* unknown status who received induction treatment in cohort 2, 445 patients were randomized between August 2015 and November 2016 to receive maintenance treatment, and all are included in the primary efficacy analysis (intent-to-treat population): FP + bevacizumab + atezolizumab (*n* = 297); FP + bevacizumab (*n* = 148; Figure 1B). Nine patients (FP + bevacizumab + atezolizumab: *n* = 4; FP + bevacizumab: *n* = 5) did not receive any study treatment and are not included in the safety analysis. A summary of baseline and demographic characteristics of patients enrolled in cohort 2 of the MODUL trial is presented in Table 1. There were no clinically relevant imbalances between the experimental and control arms in terms of geographic location, age, sex, cancer type, initial diagnosis, presence of lung metastases, ECOG PS, or responses seen at the end of induction treatment.

**Table 1:** Summary of baseline and demographic characteristics at randomization: cohort 2 (first-line *BRAF*^*wt*^ patients)

Characteristic	Fluoropyrimidine + bevacizumab + atezolizumab (*n* = 297)	Fluoropyrimidine + bevacizumab (*n* = 148)
Geographic location, *n* (%)^a^		
Europe	266 (89.6)	132 (89.2)
Americas	23 (7.7)	10 (6.8)
Africa	3 (1.0)	2 (1.4)
Asia	5 (1.7)	4 (2.7)
Response at end of induction treatment, *n* (%)^b^		
CR/PR	187 (63.0)	88 (59.5)
SD	109 (36.7)	60 (40.5)
Median age, years (range)	62.0 (25-87)	62.0 (27-83)
Age category, years, *n* (%)		
18-64	175 (58.9)	79 (53.4)
65-84	120 (40.4)	69 (46.6)
≥85	2 (0.7)	0
Male, *n* (%)	177 (59.6)	94 (63.5)
ECOG PS, *n* (%)		
0	173 (58.2)	93 (62.8)
1	119 (40.1)	53 (35.8)
>1	5 (1.7)	2 (1.4)
Cancer type, *n* (%)	*n* = 263	*n* = 131
Colon	180 (68.4)	89 (67.9)
Rectal	83 (31.6)	42 (32.1)
Sites of metastatic disease, *n* (%)		
Liver	234 (78.8)	111 (75.0)
Lung	140 (47.1)	64 (43.2)
Initial diagnosis, *n* (%)	*n* = 291	*n* = 145
Synchronous	221 (75.9)	115 (79.3)
Metachronous	70 (24.1)	30 (20.7)
**Baseline biomarker status**		
*BRAF* mutation status unknown, *n*	10	3
*KRAS* mutation status, *n* (%)	*n* = 282	*n* = 139
Mutant	156 (55.3)	78 (56.1)
Wild-type	126 (44.7)	61 (43.9)
*NRAS* mutation status, *n* (%)	*n* = 251	*n* = 119
Mutant	13 (5.2)	11 (9.2)
Wild-type	238 (94.8)	108 (90.8)
Tumor location, *n* (%)	*n* = 263	*n* = 131
Right	56 (21.3)	25 (19.1)
Left	207 (78.7)	106 (80.9)
Microsatellite stability status, *n* (%)	*n* = 252	*n* = 126
MSI	5 (2.0)	2 (1.6)
MSS	247 (98.0)	124 (98.4)

CR, complete response; ECOG PS, Eastern Cooperative Oncology Group performance status; MSI, microsatellite instable; MSS, microsatellite stable; PR, partial response; SD, stable disease.

a See Supplementary Table S2, available at https://doi.org/10.1016/j.esmoop.2022.100559, for full list of participating sites and countries.

b One patient was re-classified to progressive disease during data cleaning.

The biomarker status of the patients’ tumors at the time of randomization into maintenance treatment was also well balanced between the experimental and control arms (Table 1). *KRAS* and *NRAS* mutation status prevalence was as expected in the first-line setting, as were tumor location and microsatellite stability status. There was a slightly higher proportion of patients with *BRAF* unknown status in the experimental arm.

Treatment duration (median) during induction was 4.1 months in both arms. All patients who went on to be randomized to maintenance therapy achieved disease control at the end of induction therapy (CR/PR, 62%; SD, 38%). During the maintenance phase, treatment duration was 6.2 months with atezolizumab, and 5.5 months in the control arm. The main reasons for drug discontinuation in the experimental versus control arms, respectively, during maintenance at 2 years’ follow-up (data cut-off 31 May 2019) were: disease progression (60.6% versus 58.8%); adverse events (AEs; 14.1% versus 12.2%); physician decision (8.1% versus 11.5%); other (7.7% versus 6.1%); and withdrawal by patient (5.1% versus 6.8%).

### Efficacy

**Figure 2:**
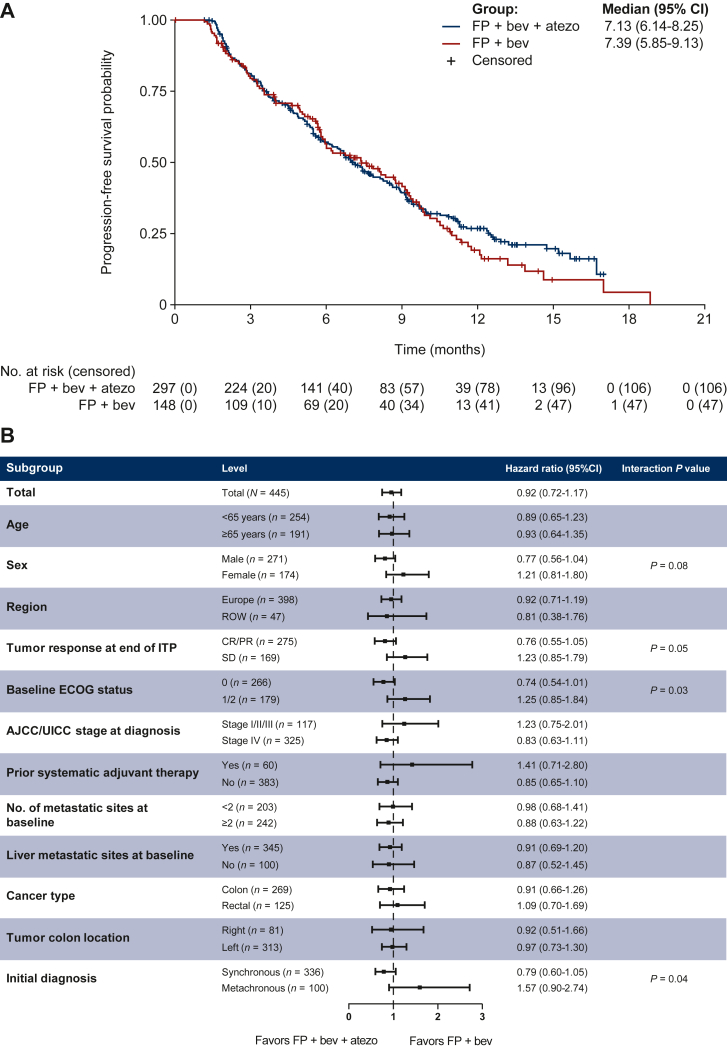

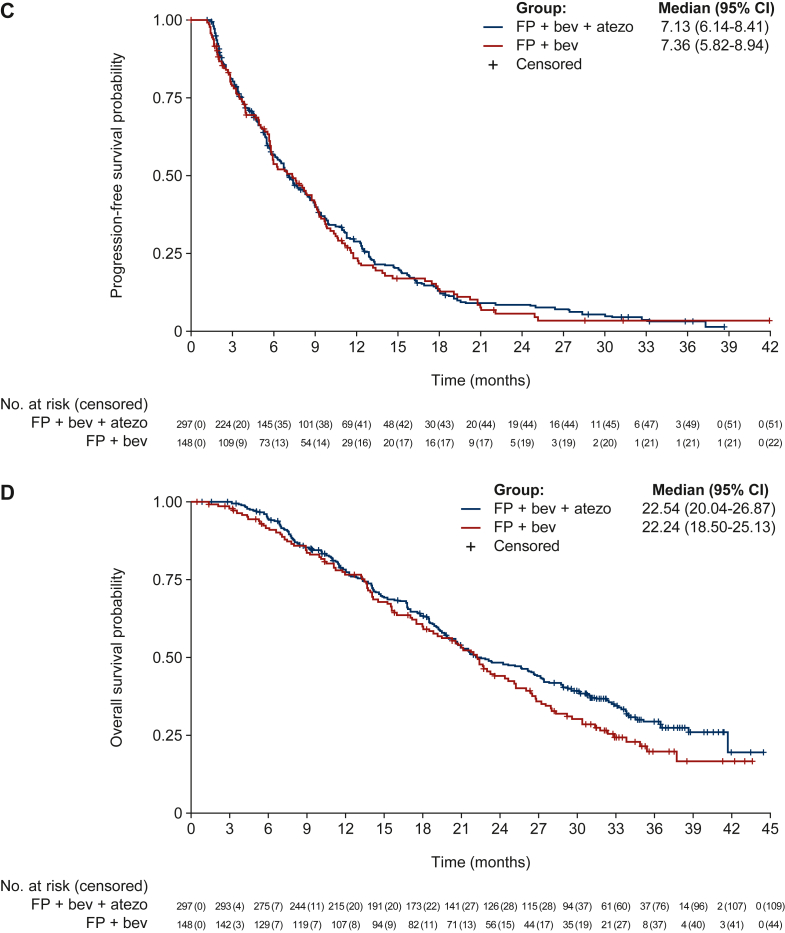
Primary analysis of progression-free survival in cohort 2 (first-line *BRAF*^*wt*^ patients) after a median follow-up of 10.5 months: Kaplan–Meier curve (A) and subgroup analysis forest plot (B). Two-year analysis of long-term efficacy after a median follow-up of 20.3 months: Kaplan–Meier curves for progression-free survival (C) and overall survival (D). AJCC/UICC, American Joint Committee on Cancer/Union for International Cancer Control; atezo, atezolizumab; bev, bevacizumab; CI, confidence interval; CR, complete response; ECOG, Eastern Cooperative Oncology Group; FP, fluoropyrimidine; ITP, induction treatment population; PR, partial response; ROW, rest of the world; SD, stable disease. Median duration of induction treatment phase: 4.1 months. One microsatellite instable (MSI) patient in the FP + bev + atezo arm had a CR during the maintenance treatment phase.

A detailed presentation of efficacy outcomes from the primary analysis in May 2017 (Table 2) shows that, at a median follow-up of 10.5 months, median PFS was not improved in the experimental arm versus the control arm (HR 0.92; 95% CI 0.72-1.17; *P* = 0.483) (Figure 2A) and OS data were immature. The ORR, DCR, median TTR, and median DoR were similar in the experimental versus control arms. A planned subgroup analysis of PFS showed similar outcomes in the experimental versus control arms for most subgroups, although subgroup treatment interactions were observed for sex (male versus female), ECOG PS at baseline (0 versus 1/2), response at the end of induction treatment (CR/PR versus SD), and initial diagnosis (synchronous versus metachronous disease) (Figure 2B). In the 2-year follow-up analysis in May 2019 (with a median follow-up of 20.3 months, interquartile range 11.2-31.0 months), PFS outcome was unchanged (HR 0.95; 95% CI 0.77-1.18; *P* = 0.666) (Figure 2C) and the OS HR at the point at which nearly two-thirds of patients had an event was 0.83 (95% CI 0.65-1.05; *P* = 0.117) (Figure 2D).

**Table 2:** Overview of cohort 2 efficacy outcomes: primary analysis (May 2017)

Efficacy endpoint	Fluoropyrimidine + bevacizumab + atezolizumab (*n* = 297)	Fluoropyrimidine + bevacizumab (*n* = 148)
Median duration of follow-up, months (range)	10.6 (0.5-19.8)	10.4 (0.8-21.7)
Median progression-free survival, months (95% CI)	7.1 (6.1-8.3)	7.4 (5.9-9.1)
Hazard ratio (95% CI)	0.92 (0.72-1.17)	
Log-rank test *P* value	0.483	
Median overall survival, months (95% CI)	NE (17.9-NE)	NE (18.8-NE)
Hazard ratio (95% CI)	0.98 (0.64-1.50)	
Log-rank test *P* value	0.923	
Overall response rate, *n* (%) (95% CI)	41 (13.8) (10.1-18.3)	18 (12.2) (7.4-18.5)
Chi-square test *P* value (two-sided)	0.630	
Disease control rate, *n* (%) (95% CI)	227 (76.4) (71.2-81.1)	111 (75.0) (67.2-81.7)
Chi-square test *P* value (two-sided)	0.739	
Median duration of response, months (95% CI)	9.0 (6.1-11.2)	6.9 (4.7-7.6)
Log-rank test *P* value	0.525	
Median time to response, months (range)	4.7 (1.2-12.5)	4.1 (1.3-8.7)
ECOG PS from baseline to end of maintenance treatment phase, %		
Improved	10.4	6.8
Improved or stayed the same	79.5	85.8

*P* value (two-sided) obtained from an unstratified log-rank test.

CI, confidence interval; ECOG PS, Eastern Cooperative Oncology Group performance status; NE, not estimable.

### Safety

Data from the primary analysis in May 2017 showed that the majority of patients experienced at least one TEAE: 276 (94.2%) of those in the experimental arm and 124 (86.7%) of those in the control arm. The most common all-grade TEAEs occurring in >10% of patients were as would be expected in this setting: patients receiving FP + bevacizumab + atezolizumab experienced slightly higher rates of diarrhea, nausea, hypertension, arthralgia, and asthenia, whereas FP + bevacizumab was associated with slightly higher rates of palmar-plantar erythrodysesthesia syndrome (Table 3). The rate of grade ≥3 TEAEs was also slightly higher in the experimental arm, as was the rate of related serious TEAEs. The most common grade ≥3 TEAEs in the experimental versus control arms were hypertension, diarrhea, and palmar-plantar erythrodysesthesia syndrome. The rate of grade 5 TEAEs was not different between the two arms.

**Table 3:** Most common TEAEs^a^^,^^b^: cohort 2 primary analysis (first-line *BRAF*^*wt*^ patients)

TEAE, *n* (%)	Fluoropyrimidine + bevacizumab + atezolizumab (*n* = 293)						Fluoropyrimidine + bevacizumab (*n* = 143)					
	Grade 1	Grade 2	Grade 3	Grade 4	Grade 5	All grades	Grade 1	Grade 2	Grade 3	Grade 4	Grade 5	All grades
**Any TEAE**	41 (14.0)	125 (42.7)	100 (34.1)	7 (2.4)	3 (1.0)	276 (94.2)	28 (19.6)	53 (37.1)	39 (27.3)	3 (2.1)	1 (0.7)	124 (86.7)
Diarrhea	36 (12.3)	21 (7.2)	9 (3.1)	0	0	66 (22.5)	11 (7.7)	5 (3.5)	3 (2.1)	0	0	19 (13.3)
Nausea	43 (14.7)	11 (3.8)	2 (0.7)	0	0	57 (19.5)	18 (12.6)	6 (4.2)	0	0	0	24 (16.8)
Fatigue	31 (10.6)	14 (4.8)	1 (0.3)	0	0	46 (15.7)	15 (10.5)	6 (4.2)	1 (0.7)	0	0	22 (15.4)
Hypertension	9 (3.1)	17 (5.8)	18 (6.1)	0	0	44 (15.0)	2 (1.4)	6 (4.2)	6 (4.2)	0	0	14 (9.8)
Palmar-plantar erythrodysesthesia syndrome	21 (7.2)	18 (6.1)	3 (1.0)	0	0	42 (14.3)	9 (6.3)	11 (7.7)	5 (3.5)	0	0	25 (17.5)
Arthralgia	20 (6.8)	18 (6.1)	1 (0.3)	0	0	39 (13.3)	4 (2.8)	0	0	0	0	4 (2.8)
Vomiting	24 (8.2)	10 (3.4)	4 (1.4)	0	0	38 (13.0)	7 (4.9)	0	0	0	0	7 (4.9)
Constipation	24 (8.2)	10 (3.4)	1 (0.3)	0	0	35 (11.9)	17 (11.9)	1 (0.7)	0	1 (0.7)	0	19 (13.3)
Abdominal pain	22 (7.5)	10 (3.4)	2 (0.7)	0	0	34 (11.6)	4 (2.8)	5 (3.5)	3 (2.1)	0	0	12 (8.4)
Stomatitis	21 (7.2)	10 (3.4)	1 (0.3)	0	0	32 (10.9)	7 (4.9)	2 (1.4)	1 (0.7)	0	0	10 (7.0)
Pyrexia	23 (7.8)	7 (2.4)	2 (0.7)	0	0	32 (10.9)	10 (7.0)	3 (2.1)	0	0	0	13 (9.1)
Asthenia	16 (5.5)	15 (5.1)	0	0	0	31 (10.6)	8 (5.6)	3 (2.1)	0	0	0	11 (7.7)
Peripheral sensory neuropathy	19 (6.5)	9 (3.1)	2 (0.7)	0	0	30 (10.2)	12 (8.4)	3 (2.1)	0	0	0	15 (10.5)

TEAE(s), treatment-emergent adverse event(s).

a In ≥10% of patients in either treatment arm (all grades).

b Other grade ≥4 TEAEs not listed in the table in the fluoropyrimidine + bevacizumab + atezolizumab versus fluoropyrimidine + bevacizumab arms were: acute coronary syndrome (grade 4, 0% versus 0.7%); constipation (grade 4, 0% versus 0.7%); deep vein thrombosis (grade 4, 0.3% versus 0%); hepatic failure (grade 5, 0.3% versus 0%); hypokalemia (grade 4, 0% versus 0.7%); intestinal perforation (grade 4, 0.3% versus 0%); large intestine perforation (grade 4, 0.3% versus 0%; grade 5, 0% versus 0.7%); myocardial infarction (grade 5, 0.3% versus 0%); myocardial ischemia (grade 4, 0.3% versus 0%); respiratory failure (grade 4, 0.3% versus 0%); sepsis (grade 4, 0.7% versus 0%); septic shock (grade 5, 0.3% versus 0%); urosepsis (grade 4, 0% versus 0.7%).

Four patients experienced TEAEs with fatal outcome. These included myocardial infarction, hepatic failure, and septic shock in the experimental arm and large intestine perforation in the control arm. Two of these events were assessed as related to study treatment: hepatic failure in the experimental arm and large intestine perforation which developed after cycle 3 in the control arm, and which was assessed by the investigator as related to bevacizumab.

A greater proportion of patients in the experimental arm than in the control arm experienced a TEAE that led to a dose modification (39.6% and 27.3%, respectively; *P* = 0.0116). Patients most commonly (≥5% in either treatment arm) had a dose modification of study treatment due to MedDRA system organ class skin and subcutaneous tissue disorders (experimental arm, 6.8% versus control arm, 7.7%), general disorders and administration site conditions (8.2% versus 4.2%), gastrointestinal disorders (7.8% versus 3.5%), and infections and infestations (7.2% versus 2.1%). The most common preferred term resulting in study treatment modification in the experimental arm was palmar-plantar erythrodysesthesia syndrome (5.5%).

Immune-related TEAEs of special interest were documented as would be expected for atezolizumab, the most common any-grade events being hypothyroidism (*n* = 16, 5.5%), hyperthyroidism (*n* = 13, 4.4%), colitis (*n* = 4, 1.4%), and autoimmune hepatitis (*n* = 2, 0.7%) (Supplementary Table S2, available at https://doi.org/10.1016/j.esmoop.2022.100559).

When safety was evaluated at the 2-year update in May 2019, no major differences in the rate or profile of TEAEs were observed in either treatment arm (Supplementary Table S3, available at https://doi.org/10.1016/j.esmoop.2022.100559).

## Discussion

Induction therapy for up to 4-6 months followed by continued maintenance therapy, but with fewer cytotoxic agents (de-escalation), is commonplace in mCRC to ensure that cumulative toxicity does not occur.^1^^,^^2^ The post-induction ‘maintenance’ setting is unique in patients with mCRC as it is characterizable both clinically (relatively low tumor burden) and molecularly (the presence of aggressive clones that could not have been eliminated by induction chemotherapy), and offers an excellent setting in which patients have not exhausted all therapeutic options and do not have important cumulative toxicities. Switching treatment regimens between the induction and maintenance phases to include new targeted agents takes advantage of the window of opportunity the maintenance phase provides to test for clinical efficacy signals before resistance to standard chemotherapies occurs.^10^

MODUL is the largest randomized umbrella maintenance study in the first-line mCRC setting and the largest chemo-immunotherapy study in first-line mCRC reported to date. The MODUL trial includes an active common control backbone of FP + bevacizumab for all maintenance arms, independent of the cohort and experimental treatment, which has been established as a standard of care in two phase III trials.^4^^,^^5^ However, we acknowledge that this concept is debatable as the findings of a recent meta-analysis concluded that shared decision making should include observation as an acceptable maintenance strategy given the lack of significant OS benefit with an FP ± bevacizumab in this setting.^16^ The inclusion of a common control arm across all MODUL cohorts permits comparison between experimental treatments and also circumvents the recruitment issues suffered with other biomarker-driven trials, as evidenced by brisk accrual to the screening portion of the trial and completion of cohort 2.

In the primary analysis of cohort 2 conducted at an overall median follow-up of 10.5 months, some separation favoring FP + bevacizumab + atezolizumab (experimental arm) was apparent in the Kaplan–Meier plot of PFS after ∼10 months, but a statistically significant difference in PFS was not shown. Similarly, no significant differences were detected with respect to OS, although these analyses were limited by low event rates and immaturity of the data. In the 2-year follow-up analysis (with a median follow-up of 20.3 months), there was no improvement in PFS or OS with the addition of atezolizumab to FP/bevacizumab in the *BRAF*^*wt*^ first-line maintenance mCRC population. Secondary ORR, DCR, DoR, and TTR endpoints were each numerically higher in the experimental arm, although these differences were not statistically significant when tested.

Although detrimental treatment effects in subgroups were observed for males versus females (HR 0.77 versus 1.21), an ECOG PS of 0 versus 1/2 (HR 0.74 versus 1.25), tumor response of CR/PR versus SD at the end of induction treatment (HR 0.76 versus 1.23), and an initial diagnosis of synchronous versus metachronous disease (HR 0.79 versus 1.57), it is important to note that the interaction test analysis was not powered to show statistical significance. It is also worth acknowledging that subgroup analyses can pose multiplicity concerns, with multiple subgroup testing leading to false-positive results by chance alone. As a result, the interaction tests and associated *P* values should be considered as descriptive only.

There were some unexpected patient characteristics, but no clear differences that would be expected to impact efficacy outcomes. MSI-like tumors are known to have the highest expression of *PD-L1* and *PD-L2* genes, and therefore respond well to agents blocking the programmed cell death protein 1/PD-L1 pathway,^11^ such as atezolizumab. However, many MSI tumors are *BRAF* mutated^17^^,^^18^ and most of them were assigned to other cohorts in MODUL.

The safety profile of atezolizumab + FP/bevacizumab observed in MODUL cohort 2 is consistent with previous findings. Overall, AEs observed during both the induction and maintenance treatment phases of the study appeared manageable and were consistent with the known safety profile of the study treatments with no new safety signals identified. As would be expected, the addition of atezolizumab to the maintenance regimen resulted in an increase in AEs including grade ≥3 events and SAEs. Notably, the increase in the incidences of diarrhea, vomiting, rash, arthralgia, hypertension, hypothyroidism, and hyperthyroidism with the addition of atezolizumab was mostly due to grade ≤2 events. Despite the increase in safety events associated with atezolizumab, treatment exposure and duration were higher in patients who received it in addition to bevacizumab and FP with a notable difference in the number of patients continuing treatment beyond 24 cycles. Furthermore, the addition of atezolizumab to a standard maintenance regimen did not appear to markedly alter the occurrence of AEs considered related to bevacizumab as the most frequent (≥4% of patients) investigator-assessed bevacizumab-related AEs (hypertension, epistaxis, nausea, and fatigue) were the same in both treatment arms.

We would like to acknowledge some potential limitations of the MODUL study. Firstly, its innovative, open-label, signal-seeking, exploratory design was chosen in order to help with the identification and development of new drug combinations for use in the maintenance setting without the need for large, randomized, placebo-controlled trials. Nevertheless, the study design worked well in principle and should be considered in the future to accelerate screening for active regimens in advanced CRC, in particular when coupled with more detailed circulating tumor DNA analyses. Limitations included no blinding of patients receiving experimental therapy (in this case atezolizumab) in addition to standard of care (FP + bevacizumab) and no placebo arm in any of the cohorts. Furthermore, MODUL was not intended to be a registrational study or result in any new applications to health authorities.

Secondly, when MODUL was initiated, it was felt that introducing the immune-stimulating combination of atezolizumab + FP + bevacizumab as maintenance therapy at the time patients with *BRAF*^*wt*^ mCRC were thought to be most likely to have their lowest tumor burden (i.e. after achieving an objective response with first-line chemotherapy) would be beneficial. Unfortunately, this approach did not work as patients with predominantly MSS CRC do not appear to be immune engaged. Furthermore, any aggressive or resistant clones remaining after standard induction therapy did not appear to be good candidates for maintenance therapy with atezolizumab + FP + bevacizumab, although we were unable to examine this hypothesis further as biomarker data were not collected after induction therapy. The addition of immunotherapy may still have a role in patients with a higher tumor burden, a concept that has been explored in the randomized phase II AtezoTRIBE study. Findings from this study showed that the addition of atezolizumab to upfront FOLFOXIRI/bevacizumab and subsequent maintenance therapy prolonged PFS in patients with molecularly unselected mCRC, although there was no difference in RECIST response rate.^19^ In the MSS or proficient mismatch repair subgroup (*n* = 199), PFS was also longer with FOLFOXIRI/bevacizumab + atezolizumab (12.9 versus 11.4 months; HR 0.78; 80% CI 0.62-0.97; *P* = 0.071).^19^ OS data for AtezoTRIBE were immature at the time of the presentation and further results from this study are awaited with interest. In parallel, results are starting to emerge from studies of other strategies designed to increase the susceptibility of MSS mCRC to immunotherapy;^20^^,^^21^ these will be considered alongside the MODUL cohort 4 results which are to be reported separately.

In conclusion, adding atezolizumab to FP/bevacizumab as first-line maintenance treatment for patients with *BRAF*^*wt*^ mCRC did not lead to improvement in efficacy outcomes. While the results are disappointing, findings from cohort 2 of the MODUL trial add to the body of evidence indicating that immunotherapy has very limited efficacy in patients with MSS mCRC, despite clear activity in patients with MSI cancers. It is clear that further efforts are required to find new strategies to circumvent the complex underlying immune escape mechanisms in patients with MSS CRC. More comprehensive biomarker studies that will include additional metastatic tumor and plasma biomarkers from MODUL patients are ongoing and will be presented.
