# Supplementary material for: MODUL cohort 2: an adaptable, randomized, signal-seeking trial of fluoropyrimidine plus bevacizumab with or without atezolizumab maintenance therapy for BRAFwt metastatic colorectal cancer
Source: ESMO Open. 2022 Aug 24;7(5):100559. doi: 10.1016/j.esmoop.2022.100559 (PMC9588902; doi:10.1016/j.esmoop.2022.100559)
Supplement: Supplementary Material [file mmc1.docx]

**SUPPLEMENTARY MATERIAL**

**Table S1** List of active study countries, and investigators for the MODUL trial 2

**Table S2** Immune-related TEAEs (any grade) of special interest: Cohort 2 primary analysis (first-line BRAF^wt^ patients) 9

**Table S3** Most common TEAEs: Cohort 2 2-year analysis (May 2019) 10

**Table S1** List of active study countries, and investigators for the MODUL trial

| **Investigator name** | **Site name** | **Country** | **Number of patients enrolled** |
| --- | --- | --- | --- |
| Siena, Salvatore | Asst Grande Ospedale Metropolitano Niguarda | Italy | 31 |
| Nasti, Guglielmo | Irccs Istituto Nazionale Tumori Fondazione Pascale | Italy | 28 |
| Van Cutsem, Eric | Uz Leuven Gasthuisberg | Belgium | 21 |
| Frassineti, Giovanni | Irst Istituto Scientifico Romagnolo Per Lo Studio E Cura Dei Tumori | Italy | 21 |
| Bragagnoli, Arinilda | Hospital De Cancer De Barretos | Brazil | 20 |
| Streb, Joanna | Szpital Uniwersytecki W Krakowie | Poland | 19 |
| Höhler, Thomas | ProsperHospital, Medizinische Klinik I | Germany | 18 |
| Kalofonos, Haralabos | University Hospital of Patras | Greece | 17 |
| Lonardi, Sara | Irccs Istituto Oncologico Veneto | Italy | 16 |
| Di Bartolomeo, Maria | Irccs Istituto Nazionale Dei Tumori IntSC | Italy | 16 |
| Martens, Uwe | SlkKliniken Heilbronn Gmbh Klinik Fur Innere Medizin Iii | Germany | 15 |
| Al Batran,Salah-Eddin | Krankenhaus Nordwest Klinik F Onkologie Und Hamatologie | Germany | 14 |
| Illmer,Thomas | Bag FreibergRichter, Jacobasch, Illmer, Wolf Gemeinschaftspraxis | Germany | 14 |
| Quintela, Antonio | Hospital De Santa Maria | Portugal | 14 |
| Tabernero, Josep | Hospital Univ Vall D’Hebron | Spain | 14 |
| Andre, Thierry | Hopital Saint Antoine | France | 13 |
| Basso, Michele | Policlinico Universitario Agostino Gemelli UOC | Italy | 13 |
| Jungic, Sasa | University Clinical Center Of The Republic Of Srpska | Bosnia & Herzegovina | 13 |
| Samuel, Leslie | Aberdeen Royal Infirmary | UK | 12 |
| Maiello, Evaristo | Irccs Ospedale Casa Sollievo | Italy | 12 |
| Hendlisz, Alain | Institut Jules Bordet X | Belgium | 12 |
| Gonzalez Trujillo, Jose Luis | Fundacion Rodolfo Padilla Padilla AC | Mexico | 12 |
| Kröning, Hendrik | Onkologische Gemeinschaftspraxis | Germany | 12 |
| Kim, Tae Won | Asan Medical Center | South Korea | 12 |
| Chau, Ian | Royal Marsden Hospital | UK | 11 |
| Ball, Simon | Queen’s Hospital | UK | 11 |
| Karthaus, Meinolf | Stadt Klinikum Munchen Gmbh Klinikum Neuperlach Klinik Fur Hamatologie Und Onkologie | Germany | 11 |
| George, Nicol | Broomfield Hospital | UK | 11 |
| Girotto, Gustavo | Faculdade De Medicina De Sao Jose Do Rio | Brazil | 10 |
| Gorini, Carlos | Hospital Nossa Senhora | Brazil | 10 |
| Heinrich, Bernhard | Hamatologisch Onkologische Praxis Dr Med Heinrich | Germany | 10 |
| Liersch, Rüdiger | Gemeinschaftspraxis Fur Hamatologie Und Onkologie | Germany | 10 |
| Müller-Huesmann, Harald | Bruderkrankenhaus St Josef | Germany | 10 |
| Yalcin, Suayib | Hacettepe Uni Medical Faculty Hospital | Turkey | 10 |
| Cicin, Irfan | Trakya University Medical Faculty Research And Practice Hospital | Turkey | 10 |
| Zampino, Giulia | Irccs Istituto Europeo Di Oncologia Ieo | Italy | 10 |
| Harrison, Mark | Mount Vernon Hospital | UK | 10 |
| Hernandez, Carlos Alberto | Oaxaca Site Management Organization | Mexico | 10 |
| Tjulandin, Sergei | Russian Oncology Research Center NA NN Blokhin | Russia | 10 |
| Popova, Ekaterina | Bashkirian Republican Clinical Oncology Dispensary | Russia | 10 |
| de Graaf, J.C. | Isala Klinieken | Netherlands | 9 |
| Ross, Paul | Guys And St Thomas NHS Foundation Trust, Guy’s Hospital | UK | 9 |
| Guimbaud, Rosine | Hopital Rangueil | France | 9 |
| Ahn, Joong Bae | Severance Hospital, Yonsei University Health System | South Korea | 9 |
| Fountzilas, George | Euromedical General Clinic Of Thessaloniki | Greece | 9 |
| Creemers, Geert Jan M. | Catharina Zkhs Inwendige Geneeskunde Afd | Netherlands | 8 |
| Baijal, Shobhit | Birmingham Heartlands Hospital | UK | 8 |
| Mineur, Laurent | Clinique Sainte Catherine | France | 8 |
| Metges, Jean-Philippe | Hopital Augustin Morvan Federation De Cancerologie | France | 8 |
| Pfeiffer, Per | Odense Universitetshospital | Denmark | 8 |
| Salud Salvia, Antonia | Hospital Universitari Arnau De Vilanova De Lleida | Spain | 8 |
| Rodríguez Salas, Nuria | Hospital Universitario La Paz | Spain | 8 |
| El Serafy, Mustafa | National Cancer Institute | Egypt | 8 |
| Yavuz, Sinan | Acibadem University School Of Medicine, Adana Hospital | Turkey | 8 |
| Pinczowski, Helio | Instituto De Ensino E Pesquisa Sao Lucas Iep | Brazil | 7 |
| Venerito,Marino | Universitatsklinikum Magdeburg Klinik Fur Gastroenterologie Und Hepatologie | Germany | 7 |
| Mavroudis, Dimitris | Univ General Hosp Heraklion | Greece | 7 |
| Sgouros, Joseph | Agioi Anargyroi 3rd | Greece | 7 |
| Iveson, Tim | Southampton General Hospital | UK | 7 |
| Jary, Marine | Hopital Jean Minjoz | France | 7 |
| Garcia Carbonero, Rocio | Hospital Universitario 12 De Octubre | Spain | 7 |
| Lazaretti, Nicolas Silva | Hospital Da Cidade De Passo Fundo Centro De Pesquisa Em Oncologia | Brazil | 6 |
| Hunting, Jarmo | St Antonius Locatie Leidsche Rijn | Netherlands | 6 |
| Pereira, Rodrigo | Hospital Das Clinicas Ufrgs | Brazil | 6 |
| Meiler, Johannes | Mvz Fur Hamatologie, Onkologie, Strahlentherapie Und Palliativmedizin Klinik Dr Hancken | Germany | 6 |
| Hoeffkes, Heinz-Gert | Klinik Fulda, Medizinisches Versorgungszentrum Osthessen Gmbh | Germany | 6 |
| Moosmann, Nicolas | Krankenhaus Barmherziger Bruder Klinik Fur Internistische Onkologie Hamatologie | Germany | 6 |
| Decker, Thomas | 271973 Prof. Dr. med. Thomas Decker | Germany | 6 |
| Kubicka, Stefan | Klinikum Am Steinenberg Ermstalklinik | Germany | 6 |
| Van Laethem, Jean-Luc | Hospital Erasme | Belgium | 6 |
| Gil Calles, Silvia | Hospital Regional Universitario Carlos Haya | Spain | 6 |
| Guillen Ponce, Carmen | Hospital Ramon Y Cajal | Spain | 6 |
| Salgado Fernandez, Mercedes | Complejo Hospitalario De Orense | Spain | 6 |
| Karimi, Masoud | Karolinska Hospital Oncology Radiumhemmet | Sweden | 6 |
| Stübs, Patrick | Drk Kliniken Berlin Kopenick Darmzentrum | Germany | 6 |
| Rivera Herrero, Fernando | Hospital Universitario Marques De Valdecilla | Spain | 6 |
| Mullamitha, Saifee | Christie Hospital NHS Trust | UK | 5 |
| Schnell, Roland | Pioh Pd Dr R Schnell Dr H Schulz Dr M Hellmann | Germany | 5 |
| Karabulut, Bulent | Ege Uni Medical Faculty Hospital | Turkey | 5 |
| Ducreux, Michel | Institut Gustave Roussy | France | 5 |
| Smith, Denis | Hopital Haut Leveque | France | 5 |
| Dhadda, Amandeep | Castle Hill Hospital The Queens Centre For Oncology And Haematology | UK | 5 |
| Massuti Sureda, Bartomeu | Hospital General Univ De Alicante | Spain | 5 |
| Escudero Emperador, M. Pilar | Hospital Clinico Universitario Lozano Blesa | Spain | 5 |
| Antonuzzo, Lorenzo | Azienda Ospedaliero Universitaria Careggi SC | Italy | 5 |
| Beretta, Giordano | Humanitas GavazzeniUO | Italy | 5 |
| Kim, Stefano | Ch De Montbeliard Chir Generale Digestive | France | 5 |
| Zajac, Leszek | Narodowy Instytut Onkologii Im M Sklodowskiej Curie | Poland | 5 |
| Diaz Romero, Maria del Consuelo | Instituto Nacional De Cancerologia Oncology | Mexico | 5 |
| Radosavljevic, Davorin | Institute for Oncology and Radiology of Serbia | Serbia | 5 |
| Andric, Zoran | Clinical Center Bezanijska Kosa | Serbia | 5 |
| Grootscholten, Cecile (M.I) | Antoni Van Leeuwenhoek Ziekenhuis | Netherlands | 5 |
| Park, Joon Oh | Samsung Medical Center | South Korea | 5 |
| Han, Sae Won | Seoul National University Hospital | South Korea | 5 |
| Dominguez, Adriana | Cancerologia De Queretaro Oncologia | Mexico | 4 |
| Bauer, Stefan | Gemeinschaftspraxis Onkologisches Zentrum Lebach Caritas Krankenhaus Lebach | Germany | 4 |
| Yumuk, Fulden | Marmara Uni Faculty of Medicine | Turkey | 4 |
| Pentheroudakis, George | University Hospital of Ioannina | Greece | 4 |
| Tougeron, David | Chu La Miletrie Gastro Enterologie Endoscopies | France | 4 |
| Ben Abdelghani, Meher | Chu De Strasbourg Icans | France | 4 |
| Skuladottir, Halla | Regionshospitalet Herning Onkologisk Afdeling | Denmark | 4 |
| Safont Aguilera, M. Jose | Hospital General Universitario De Valencia | Spain | 4 |
| Gallego Plazas, Javier | Hospital General Universitario De Elche | Spain | 4 |
| Guillot Morales, Monica | Hospital Universitario Son Espases | Spain | 4 |
| Aranda Aguilar, Enrique | Hospital Universitario Reina Sofia | Spain | 4 |
| Dueñas Garcia, Rosario | Complejo Hospitalario De Jaen Hospital Universitario Medico Quirurgico | Spain | 4 |
| Garcia Alfonso, Pilar | Hospital General Universitario Gregorio Maranon | Spain | 4 |
| Stein, Alexander | Universitatsklinikum HamburgEppendorf, Onkologisches Zentrum, Studienzentrale Der Ii Med Klinik | Germany | 3 |
| Schwaner, Ingo | Onkologische Schwerpunktpraxis Kurfurstendamm | Germany | 3 |
| Strumberg, Dirk | Klinik Der Ruhr University Bochum Marien Hospital Herne | Germany | 3 |
| Kowalyszyn, Rubén Dario | Clinica Viedma | Argentina | 3 |
| Kaen, Diego | Centro Oncologico Riojano Integral Cori | Argentina | 3 |
| Ruiz Casado, Ana | Hospital Universitario Puerta De Hierro | Spain | 3 |
| Jensen, Benny | Herlev Hospital Afdeling For Krftbehandling | Denmark | 3 |
| Barros, Anabela | Huc Servico De Oncologia Medica | Portugal | 3 |
| Jimenez Fonseca, Paula | Hospital Univ Central De Asturias | Spain | 3 |
| Negri, Francesca | AO Universitaria Di Parma | Italy | 3 |
| Boukovinas, Ioannis | Bioclinic Thessaloniki | Greece | 3 |
| Vatansever, Sezai | Istanbul Uni Capa Medical Faculty Institute Of Oncology | Turkey | 3 |
| Ocvirk, Janja | Institute of Oncology Ljubljana | Slovenia | 3 |
| Decaestecker, Jochen | Az Delta Campus Rumbeke | Belgium | 3 |
| Trajkovic-Vidakovic, Marija | Albert Schweitzer Ziekenhuis Loc Dordrecht | Netherlands | 3 |
| Mattos, Ederson | Hospital Amaral Carvalho | Brazil | 2 |
| Vogel, Arndt | Medizinische Hochschule Zentrum Innere Medizin Abt Hamatologie U Onkologie | Germany | 2 |
| Graeven, Ullrich | Kliniken Maria Hilf Gmbh, Krankenhaus St Franziskus | Germany | 2 |
| Stenfatt Larsen, Jim | Sygehus Syd Roskilde Onkologisk Haematologisk Ambulatorium | Denmark | 2 |
| Turpin, Anthony | Hopital Claude Huriez | France | 2 |
| Petorin, Caroline | Chu Estaing Chir Generale Digestive A Et B | France | 2 |
| Iseas, Soledad | Hospital De Gastroenterologia Dr Bonorino Udaondo | Argentina | 2 |
| Manzano Mozo, Jose Luis | Hospital Universitari Germans Trias I Pujol | Spain | 2 |
| Lopez Lopez, Rafael | Complejo Hospitalario Universitario De Santiago Chus | Spain | 2 |
| Lacasta Muñoa, Adelaida | Hospital De Donostia Servicio De Oncologia Medica | Spain | 2 |
| El Bassiouny, Mohamed | Ain Shams University Hospital | Egypt | 2 |
| Milella, Michele | AOUI Verona Ospedale Policlinico GB Rossi Borgo Roma | Italy | 2 |
| Killing, Birgitta | Klinikum Wetzlar Braunfels, Klinik Fur Hamatologie Onkologie Und Palliativmedizin | Germany | 2 |
| Afonso Gomez, Ruth | Complejo Hospitalario Nuestra Senora De La Candelaria | Spain | 2 |
| Vera, Ruth | Hospital De Navarra | Spain | 2 |
| De Gramont, Aimery | Institut Hospitalier Franco Britannique Cancerologie | France | 2 |
| Johnsson, Anders | Skanes University Hospital | Sweden | 2 |
| Houbiers, Ghislain | Chc Montlegia | Belgium | 2 |
| Borchert, Kersten | Klinikum Magdeburg Ggmbh Klinik Fur Allgemein Und Viszeralchirurgie | Germany | 1 |
| Mahlberg, Rolf | Klinikum Mutterhaus Der Borromaeerinnen Ggmbh Haematologie Onkologie | Germany | 1 |
| Alonso Orduña, Vicente | Hospital Universitario Miguel Servet | Spain | 1 |
| Aparicio Urtasun, Jorge | Hospital Universitario La Fe | Spain | 1 |
| Nørgaard Petersen, Lone | Rigshospitalet Onkologisk Klinik | Denmark | 1 |
| Zeuli, Massimo | Istituto Regina Elena | Italy | 1 |
| Phoutthasang, Valerie | Hopital Caremeau Gastro Enterologie | France | 1 |
| Salek, Tomas | Narodny Onkologicky Ustav Oddelenie Klinickej Onkologie E | Slovakia | 1 |
| Kakalejcik, Marian | Poko Poprad | Slovakia | 1 |
| Chau, Ian | Royal Marsden Hospital | UK | 1 |
| Jacobs, Georg | Praxis Fur Hamatologie Onkologie | Germany | 1 |
| Imholz, A.L.T. | Deventer Ziekenhuis Interne Geneeskunde | Netherlands | 1 |

**Table S2** Immune-related TEAEs (any grade) of special interest: Cohort 2 primary analysis (first-line *BRAF^wt^* patients)

| **TEAE, *n* (%)** | **Fluoropyrimidine + bevacizumab + atezolizumab (*n*=293)** | **Fluoropyrimidine + bevacizumab (*n*=143)** |
| --- | --- | --- |
| Hypothyroidism | 16 (5.5) | 0 |
| Hyperthyroidism | 13 (4.4) | 0 |
| Thyroiditis | 1 (0.3) | 0 |
| Autoimmune hepatitis | 2 (0.7) | 0 |
| Colitis | 4 (1.4) | 0 |
| Autoimmune colitis | 1 (0.3) | 0 |
| Pneumonitis | 1 (0.3) | 0 |
| Keratitis | 1 (0.3) | 0 |
| Uveitis | 1 (0.3) | 0 |

TEAE, treatment-emergent adverse event.

**Table S3** Most common TEAEs^a,b^: Cohort 2 2-year analysis (May 2019)

| **TEAE, *n* (%)** | **Fluoropyrimidine + bevacizumab + atezolizumab (*n*=293)** | | | | | |  | **Fluoropyrimidine + bevacizumab (*n*=143)** | | | | | | |
| --- | --- | --- | --- | --- | --- | --- | --- | --- | --- | --- | --- | --- | --- | --- |
|  | **Grade 1** | **Grade 2** | **Grade 3** | **Grade 4** | **Grade 5** | **All grades** |  | **Grade 1** | | **Grade 2** | **Grade 3** | **Grade 4** | **Grade 5** | **All grades** |
| **Any TEAE** | 33 (11.3) | 123 (42.0) | 112 (38.2) | 8 (2.7) | 4 (1.4) | 280 (95.6) |  | 24 (16.8) | | 54 (37.8) | 42 (29.4) | 5 (3.5) | 1 (0.7) | 126 (88.1) |
| Diarrhea | 40 (13.7) | 28 (9.6) | 12 (4.1) | 0 | 0 | 80 (27.3) |  | 13 (9.1) | | 6 (4.2) | 3 (2.1) | 0 | 0 | 22 (15.4) |
| Nausea | 46 (15.7) | 15 (5.1) | 3 (1.0) | 0 | 0 | 65 (22.2) |  | 19 (13.3) | | 8 (5.6) | 0 | 0 | 0 | 27 (18.9) |
| Vomiting | 26 (8.9) | 11 (3.8) | 4 (1.4) | 0 | 0 | 41 (14.0) |  | 8 (5.6) | 0 | | 0 | 0 | 0 | 5 (5.6) |
| Abdominal pain | 25 (8.5) | 12 (4.1) | 2 (0.7) | 0 | 0 | 39 (13.3) |  | 4 (2.8) | 6 (4.2) | | 3 (2.1) | 0 | 0 | 13 (9.1) |
| Constipation | 27 (9.2) | 11 (3.8) | 1 (0.3) | 0 | 0 | 39 (13.3) |  | 16 (11.2) | 2 (1.4) | | 0 | 1 (0.7) | 0 | 19 (13.3) |
| Stomatitis | 22 (7.5) | 11 (3.8) | 3 (1.0) | 0 | 0 | 36 (12.3) |  | 6 (4.2) | 3 (2.1) | | 2 (1.4) | 0 | 0 | 11 (7.7) |

TEAE(s), treatment-emergent adverse event(s).

^a^In ≥10% of patients in either treatment arm (all grades). ^b^Other grade ≥4 TEAEs not listed in the table in the fluoropyrimidine + bevacizumab + atezolizumab versus the fluoropyrimidine + bevacizumab arms were: sepsis (grade 4, 0.7 versus 0%); urosepsis (grade 4, 0 versus 0.7%); post-procedural sepsis (grade 4, 0.3 versus 0%); intestinal perforation (grade 4, 0.3 versus 0%); large intestine perforation (grade 4, 0.3 versus 0.7%); constipation (grade 4, 0 versus 0.7%); respiratory failure (grade 4, 0.3 versus 0%); deep vein thrombosis (grade 4, 0.3 versus 0%); myocardial ischemia (grade 4, 0.3 versus 0%); acute coronary syndrome (grade 4, 0 versus 0.7%); hypokalemia (grade 4, 0 versus 0.7%); neutropenia (grade 4, 0 versus 0.7%); second primary malignancy (grade 4, 0 versus 0.7%); septic shock (grade 5, 0.3 versus 0%); hepatic failure (grade 5, 0.3 versus 0%); hepatorenal failure (grade 5, 0.3 versus 0%); myocardial infarction (grade 5, 0.3 versus 0%).
